# Impact of premastectomy versus postmastectomy radiotherapy on postoperative complications in breast reconstruction: A systematic review and meta-analysis

**DOI:** 10.1016/j.jpra.2026.09.012

**Published:** 2026-09-12

**Authors:** Amy Ker, Pei-En Kao

**Affiliations:** a Department of Medical Education, Kaohsiung Chang Gung Memorial Hospital, Kaohsiung, Taiwan; b Department of Orthopaedic Surgery, Hualien Tzu Chi Hospital, Buddhist Tzu Chi Medical Foundation, Hualien, Taiwan; c School of Medicine, Chung Shan Medical University, Taichung, Taiwan

**Keywords:** Premastectomy, Postmastectomy, Radiotherapy, Breast reconstructions, Postoperative complications

## Abstract

**Background:**

The present study aimed to performed a systemic review and meta-analysis to compare. Impact of premastectomy radiotherapy (Pre-MRT) versus Postmastectomy radiotherapy (PMRT) on Postoperative Complications in Breast Reconstruction.

**Methods:**

PubMed, Web of Science, and the Cochrane Library were searched from inception to February 3, 2026. Eligible studies compared Pre-MRT and PMRT for postoperative complications following breast reconstruction. Random-effects models were used to estimate pooled risk ratios (RRs) with 95% confidence intervals (CIs).

**Results:**

Three studies, including one randomized-controlled trial and two cohort studies comprising a total of 483 breasts were included in the meta-analysis. The meta-analysis demonstrated no significant difference in the rate of postoperative complications between the PreMRT and PMRT groups (pooled RR: 1.36; 95% confidence interval [CI]: 0.90–2.06; p = 0.0583). Subgroup analyses demonstrated no statistically significant differences observed in the rates of complete flap loss, skin flap necrosis, local recurrence, hematoma, seroma, infection, implant loss, or complete nipple necrosis.

**Conclusion:**

This meta-analysis did not demonstrate a statistically significant difference in postoperative complication rates between PreMRT and PMRT in patients undergoing breast reconstruction. However, given the limited number of included studies and the imprecision of the pooled estimates, these findings should be interpreted with caution.

## Introduction

Breast cancer remains a major global public health concern, with approximately one in eight women diagnosed during their lifetime.^1^ Advances in multimodal treatment, including surgery, systemic therapy, and radiotherapy, have significantly improved survival outcomes.^1^^,^^2^ Among these, radiotherapy plays a critical role in reducing locoregional recurrence and improving long-term survival in selected patients.^3^ According to the National Comprehensive Cancer Network (NCCN) and American Society of Clinical Oncology (ASCO) Guidelines, the current standard treatment paradigm for patients with high-risk features involves mastectomy followed by postmastectomy radiotherapy (PMRT), with breast reconstruction performed either immediately or in a delayed fashion.^4^^,^^5^ Based on the evidence from the EBCTCG meta-analysis^6^^,^^7^ established that PMRT significantly reduces both locoregional recurrence and breast cancer mortality, forming the foundation of current treatment guidelines.

Despite its established oncologic benefits, the integration of PMRT with breast reconstruction remains challenging. Exposure of reconstructed tissues to radiation has been associated with an increased risk of complications, including capsular contracture, flap-related complications, and compromised cosmetic outcomes, which may ultimately affect patient satisfaction and quality of life.^8^, ^9^, ^10^ As a result, optimizing the sequencing of radiotherapy and reconstruction has become an important clinical consideration.

Premastectomy radiotherapy (PreMRT) has recently emerged as an alternative sequencing approach to radiotherapy delivery in breast cancer. By delivering radiation to the intact breast and regional lymphatics prior to surgery, this strategy may reduce radiation-related damage to reconstructed tissues and facilitate immediate breast reconstruction.^11^ Although the current evidence remains limited and is largely based on early-phase and retrospective studies, a recent randomized clinical trial (RCT) demonstrated that PreMRT is a feasible and safe approach, with acceptable complication rates and locoregional control, while enabling timely and effective reconstruction.^11^

However, despite these promising findings, evidence comparing PreMRT and PMRT remains limited and heterogeneous, particularly with respect to patient-reported outcomes such as cosmetic satisfaction and functional recovery. Furthermore, the long-term impact of radiotherapy sequencing on these outcomes has not been well established. Therefore, this meta-analysis aimed to comprehensively evaluate and compare outcomes between PreMRT and PMRT, with a focus on patient-reported and clinical outcomes.

## Methods

This systematic review and meta-analysis was conducted in accordance with the Cochrane Handbook for Systematic Reviews of Interventions (version 6.5). and reported following the Preferred Reporting Items for Systematic Reviews and Meta-Analyses (PRISMA) guidelines.^12^ The study protocol was prospectively registered in the PROSPERO (registration number: CRD420261360579).

### Search strategy

Two investigators (AK and PEK) systematically searched PubMed, Web of Science, and the Cochrane Library from database inception to February 3, 2026. The detailed search strategies applied to each database are described below: (‘Breast cancer’ OR ‘Breast carcinoma’) AND (‘premastectomy’ OR ‘pre-mastectomy’) AND (‘postmastectomy’ OR ‘post-mastectomy’). No restrictions were placed on study design, publication date, or language. Gray literature, including conference abstracts, was also considered. Additionally, a snowballing approach was employed through manual screening of reference lists and citation tracking of included studies and relevant reviews to identify any additional eligible publications.

### Study selection and eligibility criteria

This meta-analysis was designed based on the PICO framework (Population, Intervention, Comparison, and Outcome). The components were defined as follows: P: patients with breast cancer undergoing mastectomy, radiotherapy and reconstruction surgery; I: premastectomy radiotherapy; C: postmastectomy radiotherapy; O: postoperative complication rates.

The inclusion criteria for this meta-analysis were as follows: (1) studies involving patients with breast cancer who underwent mastectomy, radiotherapy and reconstruction surgery; (2) studies comparing the complications of PreMRT and PMRT (3) a control group consisting of patients receiving PMRT (4) studies reporting data on postoperative complications; and cosmetic and functional Assessment (5) eligible study designs including cohort studies and randomized or non-RCT.

The exclusion criteria were as follows: (1) conference abstracts; (2) review articles, editorials, and letters to the editor; (3) studies without available full texts; (4) studies that did not compare PreMRT and PMRT; (5) studies that did not report complication outcomes; and (6) duplicate publications or studies with overlapping patient populations.

Two investigators (AK and PEK) independently screened the titles and abstracts of retrieved records to assess eligibility. Full texts of potentially relevant articles were subsequently reviewed according to the predefined inclusion and exclusion criteria. Any disagreements were resolved through discussion until consensus was reached.

### Data extraction and quality assessment

Data were independently extracted by two investigators (AK and PEK) from the selected studies using a standardized form. The extracted information included study design, the name of the first author, publication year, country, and sample sizes for both study and control groups. Furthermore, we recorded specific details regarding the type of surgery, main findings of each study. Statistical outcomes, such as risk estimates (incidence rates) along with their 95% confidence intervals (CIs), and postoperative complications were also documented. The methodological quality of the included studies was assessed using standardized tools according to their design. For the cohort studies, the Newcastle–Ottawa Scale (NOS) was employed,^13^ encompassing three key domains: quality of selection, comparability between groups, and outcomes of study participants. Studies scoring 7 to 9 were considered to have a low risk of bias, scores of 4 to 6 a moderate risk, and scores of <4 a high risk. For the RCT included in this review, the Cochrane Risk of Bias tool (RoB 2.0) was applied to evaluate its internal validity across five domains (randomization process, adherence to the intervention, missing outcome data, outcome measurement, and selective reporting), and then provided an overall risk of bias judgment). Any discrepancies during the extraction or assessment process were resolved through consensus or discussion.

### Primary outcome and secondary outcomes

The primary outcome was postoperative complications, including complete flap loss, skin flap necrosis, local recurrence, hematoma, seroma, infection events, implant loss and complete nipple necrosis. The secondary outcome was patient-reported outcomes after radiotherapy, evaluated using the Satisfaction with Cosmetic Outcome Questionnaire.

### Data analyses and software

All statistical analyses were conducted using R software (version 4.5.1) within the RStudio environment. For dichotomous outcomes, pooled risk ratios (RRs) with corresponding 95% CIs were calculated to compare complication rates between patients receiving PreMRT and those receiving PMRT. The evaluated complications included complete flap loss, skin flap necrosis, local recurrence, hematoma, seroma, infection, implant loss, and complete nipple necrosis. Meta-analyses were performed using the inverse-variance method under a random-effects model. Given the small number of included studies, fixed-effect models were additionally performed as sensitivity analyses to assess the robustness of the pooled estimates. A leave-one-out sensitivity analysis was also conducted for the overall postoperative complication outcome by sequentially excluding each study and recalculating the pooled effect estimate to assess the influence of individual studies on the overall result.

Between-study heterogeneity was quantified using the I² statistic.^14^ An I² value of less than 40% was interpreted as low heterogeneity, values between 40% and 75% as moderate heterogeneity, and values greater than 75% as substantial heterogeneity.

Publication bias was evaluated using funnel plot inspection when at least ten studies were available for each outcome.^15^ Statistical significance was defined as a two-sided p value < 0.05. For rare-event outcomes, studies with zero events in one treatment arm were handled using a continuity correction of 0.5 during the calculation of risk ratios. Studies with zero events in both treatment arms were excluded from the pooled risk ratio analyses because they do not contribute to the estimation of relative treatment effects.

## Results

### Included studies characteristics

A total of 128 studies were initially retrieved from three databases. After duplicate records were removed and the titles and abstracts were screened, 8 studies were considered eligible for further evaluation. Following full-text assessment, 5 studies were excluded.

Ultimately, three studies were included in the systematic review and meta-analysis, comprising one RCT and two cohort studies.^16^, ^17^, ^18^ The study selection process is illustrated in the PRISMA 2020 flow diagram (Fig. 1). The characteristics of the included studies are summarized in Table 1.

**Fig. 1:**
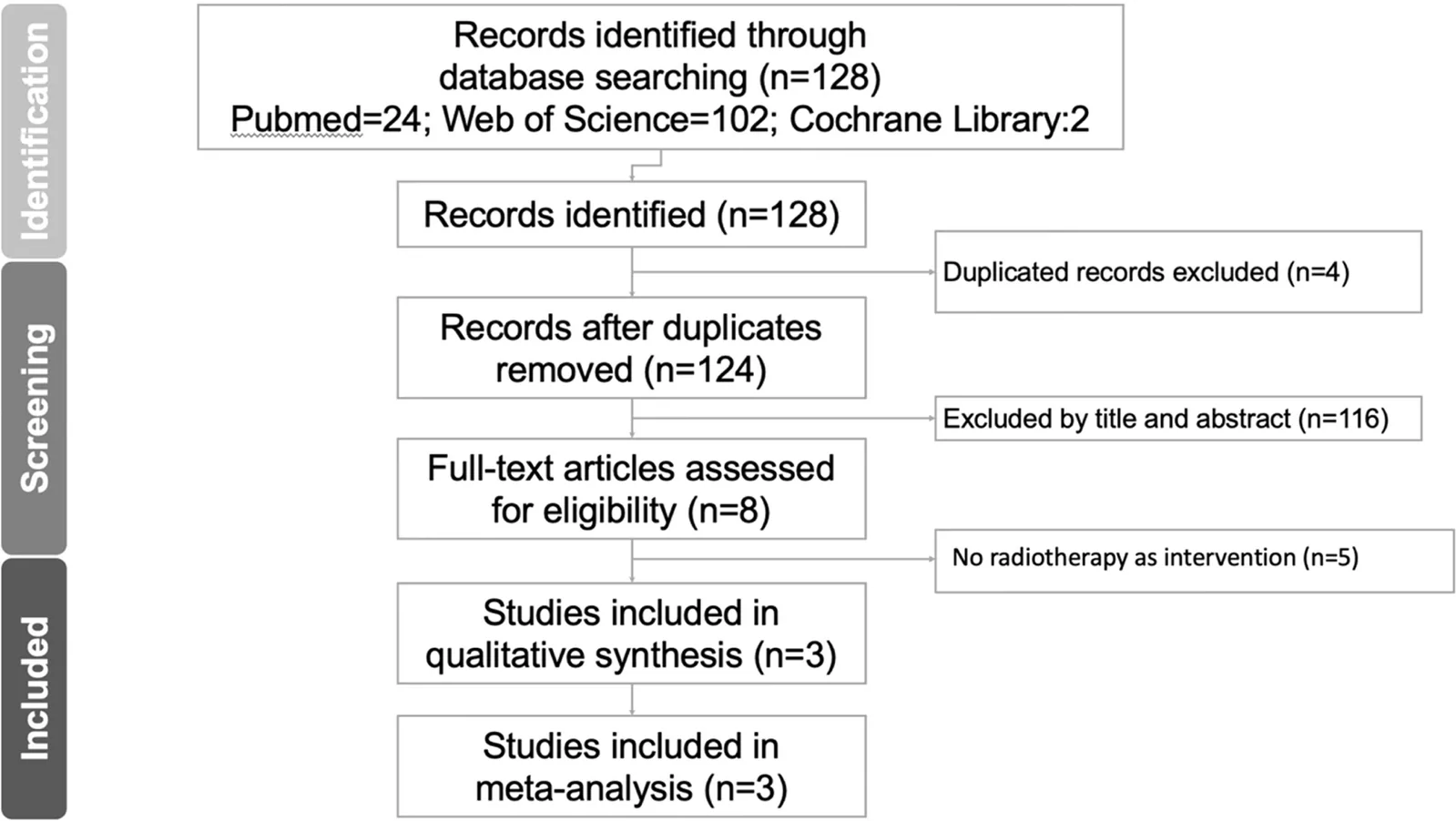
Prisma flow diagram.

**Table 1:** Characteristics of the included studies.

First Author & Year	Country	Study design	Total of participants	Total of breast	Study group	Control group	Type of Surgery	Main findings
Schaverien 2025	USA	RCT	144 participants	144 breasts	55 Pre-MRT breasts (41 cases)	153 PMRT breasts (103 cases)	1.Pre-PMRT: Skin-sparing:38 Nipple-sparing:3 Total:0 Unilateral:27 Bilateral:14 2.PMRT Skin-sparing:56 Nipple-sparing:54 Total:37 Unilateral:80 Bilateral:23	Pre-MRT with microvascular IMBR is associated with a similar complication rate to PMRT with microvascular DBR while avoiding complications relating to TE placement and a reduced time to achieve definitive breast reconstruction
Sinnott 2021	USA	RCS	369 participants	99	Pre-MRT:28 (26 cases)	PMRT:71 breasts (45 cases)	Pre-PMRT: Unilateral:146 Bilateral:223	-In prepectoral implant breast reconstruction, both pre- and postmastectomy radiation were associated with higher infection and implant loss rates than no radiation. -Postmastectomy radiation increased capsular contracture rates, comparable to premastectomy radiation, while premastectomy radiation was associated with higher seroma rates.
Sbitany 2019	USA	PCS	580 patients	176	Pre-MRT:63 breast (cohort2)	PMRT:113 breast (cohort 3)	Total skin-sparing mas- tectomy	-Pre- and postmastectomy radiation increased infection and implant/expander loss after total skin-sparing mastectomy with immediate reconstruction, with higher wound complications after preoperative radiation. -Nipple–areola complex necrosis rates were low and similar across groups, supporting the safety of nipple preservation in irradiated patients.

### Quality assessment and publication bias

The risk of bias for the included studies is summarized in Table 2. Two cohort studies.^17^^,^^18^ were assessed using the Newcastle–Ottawa Scale (NOS) and were judged to have low risk of bias, with both studies receiving 7 stars.

**Table 2:** Risk of bias of included studies using Cochrane risk of bias 2 tool for randomized controlled trials and Newcastle-Ottawa Scale for cohort studies.

Author/Year		Quality of selection		Comparability			Outcomes of study participants		
Sbitany 2019		★★★★		★			★★		
Sinnott 2021		★★★★		★			★★		
**Author/Year**	**Randomization process**		**Intervention adherence**		**Missing outcome data**	**Outcome measurement**		**Selective reporting**	**Overal RoB**
Schaverien 2025	S		S		L	L		L	S

Stars (★) were assigned in accordance with the Newcastle-Ottawa Quality Assessment Form for Cohort Studies. L: low risk of bias; RoB: risk of bias; S: some concerns.

The RCT was evaluated using the Cochrane Risk of Bias 2.0 tool (RoB 2.0) and was judged to have some concerns, primarily due to baseline imbalances and the lack of blinding of participants and personnel.^16^ However, no major issues were identified regarding deviations from intended interventions or selective outcome reporting.^16^ Given that fewer than ten studies were included in the meta-analysis, a funnel plot was not generated to evaluate publication bias.

### Postoperative complication rates

Three studies comprising a total of 483 breasts were included to evaluate the rate of postoperative complications in breast cancer patients undergoing mastectomy, radiotherapy and reconstruction.^16^, ^17^, ^18^ These studies compared postoperative complications between PreMRT and PMRT. As shown in Fig. 2, the random-effects meta-analysis demonstrated no significant difference in postoperative complications between the PreMRT and PMRT groups (pooled RR: 1.36; 95% CI: 0.90–2.06), with moderate between-study heterogeneity (I² = 64.8%). In contrast, the fixed-effect model showed a statistically significant increase in postoperative complications in the PreMRT group (RR: 1.21; 95% CI: 1.02–1.43; p = 0.0272).

**Fig. 2:**
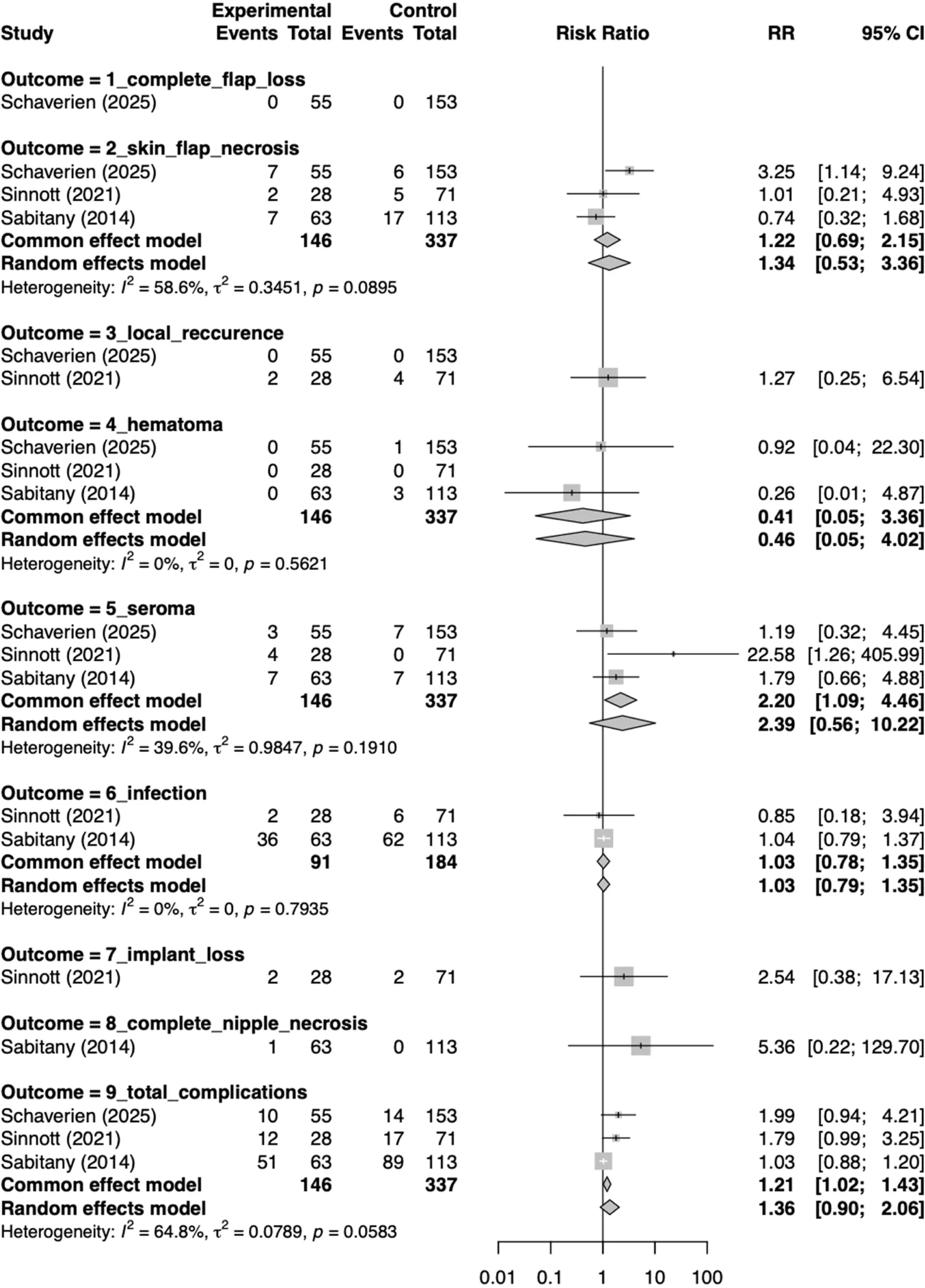
Meta-analysis of postoperative complications between the PreMRT and PMRT groups, stratified by complication type.

Leave-one-out sensitivity analysis showed that the pooled result remained non-significant when either Schaverien *et al.* or Sinnott *et al*. was omitted. However, after excluding Sabitany *et al*., the pooled estimate became statistically significant (RR: 1.88; 95% CI: 1.17–3.00; p = 0.0087), indicating that the overall result was sensitive to the inclusion of individual studies (Fig. 3).

**Fig. 3:**
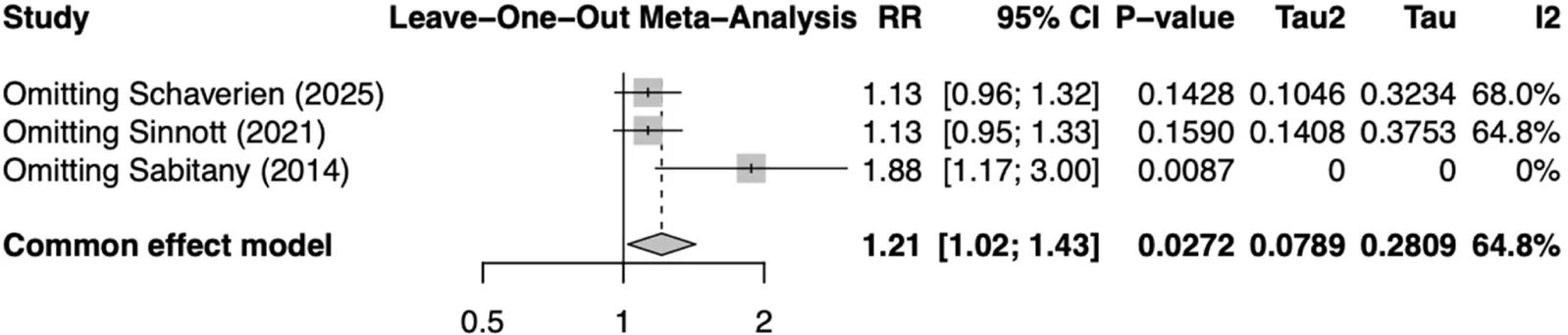
Leave-one-out sensitivity analysis of the pooled risk ratio for postoperative complications between the PreMRT and PMRT groups.

Subgroup analyses, as illustrated in Fig. 2**,** showed no statistically significant differences between the PreMRT and PMRT groups in the rates of complete flap loss, skin flap necrosis, local recurrence, hematoma, seroma, infection, implant loss, or complete nipple necrosis.

### Cosmetic and functional assessment

In *Schaverien et al.* study, when comparing Pre-MRT with PMRT groups, patient-reported outcomes showed time-dependent differences.^16^ At 6 months after radiotherapy, the PMRT-tissue expander (TE) group reported worse cosmetic outcomes than the Pre-MRT group, while no clear difference was observed between Pre-MRT and PMRT-total mastectomy (TM).

At 18 months postoperatively, functional outcomes were worse in the Pre-MRT group compared to the PMRT-TM group, suggesting a shift in outcomes over time.

## Discussion

This meta-analysis included one RCT and two cohort studies comparing the postoperative complications rate between PreMRT and PMRT.^16^, ^17^, ^18^ Current evidence did not demonstrate a statistically significant difference in postoperative complication rates between the two groups. However, the limited evidence and imprecision of the pooled estimates warrant cautious interpretation of these findings. Notably, evidence from Schaverien *et al*. suggests that patient-reported outcomes may change over time according to the timing of radiotherapy.^16^ In the early postoperative period, Pre-MRT appeared to be associated with more favorable cosmetic outcomes compared with PMRT, particularly when radiation was delivered after tissue expander placement. However, this early advantage did not necessarily persist over time. At later follow-up, functional outcomes appeared less favorable in the Pre-MRT group compared with patients receiving PMRT after mastectomy, suggesting a temporal shift in patient-reported outcomes. These findings indicate that, although overall complication rates may be comparable between Pre-MRT and PMRT, radiotherapy timing may have distinct effects on short-term cosmetic satisfaction and longer-term functional recovery. Therefore, treatment sequencing should be carefully considered when counseling patients regarding reconstruction and expected outcomes.

Radiotherapy is known to adversely affect reconstructive outcomes through several biological mechanisms. These effects can be explained by multiple underlying processes. First, radiation-induced endothelial injury leads to microvascular damage, resulting in tissue hypoxia and impaired wound healing.^19^ Second, radiation promotes fibrosis through fibroblast activation and excessive collagen deposition, leading to decreased tissue elasticity and increased tissue stiffness.^20^ Finally, radiation is associated with chronic inflammation and impaired regenerative capacity, including reduced angiogenesis and diminished cellular repair, further increasing the risk of infection and delayed healing.^19^ Consistent with these mechanisms, previous studies have demonstrated that radiotherapy is associated with impaired tissue healing and an increased risk of postoperative complications across different reconstructive approaches.^16^^,^^21^^,^^22^ However, in the present meta-analysis, no significant differences in complication rates were observed between PreMRT and PMRT. This finding should be interpreted with caution, as the available evidence remains limited, with a small number of studies, heterogeneous patient populations, and variability in treatment protocols. Therefore, further high-quality studies are warranted to better clarify the impact of radiotherapy timing on complications in breast reconstruction.

Reconstruction type represents an important determinant of postoperative outcomes in breast reconstruction. Among the included studies, two applied comparable reconstructive approaches between the PreMRT and PMRT groups, both focusing on implant-based reconstruction.^17^^,^^18^ In contrast, one study employed different reconstructive strategies across groups, with immediate implant-based reconstruction in the PreMRT and delayed breast reconstruction in the PMRT.^16^ Such heterogeneity in reconstructive timing and technique may have introduced bias and confounded comparisons between radiotherapy groups. Notably, delayed reconstruction is typically performed after the completion of radiotherapy, which may mitigate radiation-related complications compared with immediate reconstruction. Therefore, variability in reconstructive strategies across studies should be carefully considered when interpreting the impact of radiotherapy timing and may partially account for the absence of significant differences observed in the present meta-analysis.

Across the included studies, postoperative complication rates were generally comparable between PreMRT and PMRT. In a study of microsurgical breast reconstruction, no significant differences were observed in overall or major complication rates between groups, despite differences in reconstructive timing.^16^ Similarly, in prepectoral implant-based reconstruction, most complication outcomes, including infection, wound dehiscence, capsular contracture, and implant loss, were comparable between PreMRT and PMRT, with the exception of a higher rate of seroma in the PreMRT.^18^ In contrast, another study demonstrated a more heterogeneous complication profile. PreMRT was associated with increased risks of seroma, infection, incisional breakdown, and implant-related complications, whereas postmastectomy radiotherapy was more frequently associated with severe complications such as full-thickness skin necrosis and implant removal.^17^ Taken together, these findings suggest that while the overall incidence of postoperative complications may be similar between PreMRT and PMRT, the type and severity of complications may differ. This variation in complication profiles may contribute to the absence of a significant difference observed in the pooled analysis and highlights the importance of evaluating not only overall complication rates but also specific complication patterns when interpreting outcomes. This distinction may be clinically relevant when selecting treatment strategies based on patient priorities and reconstructive goals. Given the limited available evidence demonstrating no statistically significant difference in short-term postoperative complication rates between PreMRT and PMRT, radiotherapy sequencing alone should not dictate reconstructive management. The choice of reconstructive modality and timing should also be considered, as immediate versus delayed reconstruction and implant-based versus autologous reconstruction may have different susceptibility to radiation-related complications and may influence the need for subsequent revision procedures. Therefore, treatment decisions should be individualized by integrating reconstructive modality, reconstruction timing, oncologic priorities, patient characteristics, and patient preferences through multidisciplinary discussion. Such an individualized approach may help optimize reconstructive outcomes while minimizing long-term morbidity and the need for tertiary reconstructive procedures. Several important limitations inherent to the included studies merit consideration. First, substantial heterogeneity in reconstructive strategies—encompassing both reconstructive timing and surgical techniques—likely introduced treatment-related bias and may have confounded comparisons between PreMRT and PMRT groups. Second, variations in baseline patient characteristics and treatment protocols, including prior oncologic treatments, use of adjuvant therapies, and demographic differences, further limit cross-study comparability and may have influenced postoperative outcomes. Notably, although a RCT was included, imbalances in baseline characteristics and differences in reconstructive approaches between groups may compromise internal validity and limit the strength of causal inferences. In addition, the relatively small sample sizes, heterogeneity in outcome definitions, and variability in follow-up duration across studies may reduce both the precision and generalizability of the reported findings. Collectively, these limitations underscore the need for more rigorously designed studies with standardized reconstructive and oncologic protocols.

## Conclusions

In conclusion, this meta-analysis did not demonstrate a statistically significant difference in postoperative complication rates between PreMRT and PMRT in patients undergoing breast reconstruction. However, the limited number of included studies and the imprecision of the pooled estimates preclude definitive conclusions regarding the effect of radiotherapy timing. Therefore, these findings should not be interpreted as evidence of equivalence between the two treatment strategies. While the available evidence supports an individualized approach to treatment sequencing based on patient characteristics, reconstructive goals, and oncologic considerations, adequately powered, high-quality studies with standardized reconstructive and radiotherapy protocols are needed to clarify the impact of radiotherapy timing on postoperative outcomes. .

## Ethics approval and consent to participate

Not applicable.

## Consent for publication

Not applicable.

## Funding

No funding.

## Author’s contributions

Data curation: AK and PEK; formal analysis: AK and PEK; investigation: AK and PEK; methodology: AK and PEK; software: PEK; supervision: PEK; validation: AK and PEK; writing – original draft: AK; writing – review & editing: PEK. Approval of the final manuscript has been obtained from all authors prior to publication.

## Declaration of generative AI in scientific writing

During manuscript preparation, OpenAI’s ChatGPT (ChatGPT Plus, GPT-5.3) was used to assist with language refinement. Its role was limited to editorial support and did not involve study design, data analysis, interpretation of results, or the development of conclusions. All content was independently reviewed and revised by the authors, who take full responsibility for the accuracy and integrity of the manuscript.

## Clinical trial number

Not applicable.

## Competing interests

The authors declare no conflict of interest.

## Data Availability

The datasets analyzed in this research were extracted from existing literature, with all original contributors appropriately acknowledged through citations.
